# Prokaryotic RNA Associated to Bacterial Viability Induces Polymorphonuclear Neutrophil Activation

**DOI:** 10.3389/fcimb.2017.00306

**Published:** 2017-07-06

**Authors:** Nahuel Rodriguez-Rodrigues, Luis A. Castillo, Verónica I. Landoni, Daiana Martire-Greco, M. Ayelén Milillo, Paula Barrionuevo, Gabriela C. Fernández

**Affiliations:** Laboratorio de Fisiología de los Procesos Inflamatorios, Instituto de Medicina Experimental CONICET, Academia Nacional de MedicinaBuenos Aires, Argentina

**Keywords:** bacterial viability, RNA, *E. coli*, PMN, bactericidal functions

## Abstract

Polymorphonuclear neutrophils (PMN) are the first cellular line of antibacterial host defense. They sense pathogens through recognition of pathogen-associated molecular patterns (PAMPs) by innate pattern recognition receptors, such as Toll-like receptors (TLR). The aim of this study was to investigate whether PMN sense bacterial viability and explore which viability factor could be involved in this phenomenon. For this purpose, different functions were evaluated in isolated human PMN using live *Escherichia coli* (Ec) and heat-killed Ec (HK-Ec). We found that bacterial viability was indispensable to induce PMN activation, as measured by forward-scatter (FSC) increase, CD11b surface expression, chemotaxis, reactive oxygen species (ROS) generation and neutrophil extracellular trap (NET) formation. As uncapped non-polyadenylated prokaryotic mRNA has been recognized as a PAMP associated to bacterial viability by macrophages and dendritic cells, total prokaryotic RNA (pRNA) from live Ec was purified and used as a stimulus for PMN. pRNA triggered similar responses to those observed with live bacteria. No RNA could be isolated from HK-Ec, explaining the lack of effect of dead bacteria. Moreover, the supernatant of dead bacteria was able to induce PMN activation, and this was associated with the presence of pRNA in this supernatant, which is released in the killing process. The induction of bactericidal functions (ROS and NETosis) by pRNA were abolished when the supernatant of dead bacteria or isolated pRNA were treated with RNAse. Moreover, endocytosis was necessary for pRNA-induced ROS generation and NETosis, and priming was required for the induction of pRNA-induced ROS in whole blood. However, responses related to movement and degranulation (FSC increase, CD11b up-regulation, and chemotaxis) were still triggered when pRNA was digested with RNase, and were not dependent on pRNA endocytosis or PMN priming. In conclusion, our results indicate that PMN sense live bacteria through recognition of pRNA, and this sensing triggers potent bactericidal mechanisms.

## Introduction

Polymorphonuclear neutrophils (PMN) are the first cellular line of antibacterial host defense and are loaded with granules containing proteolytic enzymes and antimicrobial peptides. They follow chemotactic gradients changing their shape by spreading and are recruited in large numbers from blood adhering to and moving across the endothelium to reach infected and/or injured tissue (McDonald et al., 2010). During the first steps of PMN activation, increments in their forward scatter (FSC) occur. This has been associated with the spreading process itself, but also to the translocation and fusion of easily mobilized granules with the plasmatic membrane (exocytosis), exposing the interior membrane surface of the granule to the exterior, therefore increasing the total surface area of the cell and the FSC (Cole et al., 1995; Lacy, 2006). Additionally, exocytosis causes the up-regulation in the plasmatic membrane of molecules and receptors involved in PMN adhesion to endothelial cells, such as CD11b.

Upon encountering bacteria, PMN capture, ingest and kill them by the production of reactive oxygen species (ROS) within intracellular phagosomes (El-Benna et al., 2016). Additionally, PMN were shown to generate web-like extracellular fibers known as neutrophil extracellular traps (NETs), composed of deoxyribonucleic acid (DNA), histones, and antimicrobial granule proteins, which are highly effective at trapping and killing invasive bacteria (Amulic and Hayes, 2011). The response of PMN to bacterial structural components has been widely reported, but no studies have been performed comparing the PMN response between live and dead bacteria. The fact that bacteria stimulate differently the immune response depending on viability is plausible, considering that live vaccines trigger more vigorous immune responses than their killed counterparts (Detmer and Glenting, 2006). Moreover, protective immunization can often be achieved with a single injection of live, but not dead or attenuated microorganisms. As structural bacterial components are present in both live and killed vaccines, it is possible that dead bacteria lack certain components important to induce an effective protective immunity. In this sense, it has been demonstrated that murine bone marrow-derived dendritic cells and macrophages can directly sense microbial viability through detection of uncapped non-polyadenylated prokaryotic mRNA present only in viable bacteria (Sander et al., 2012).

Neutrophils sense pathogens through recognition of pathogen-associated molecular patterns (PAMPs) by innate pattern recognition receptors (PRP). Although some receptors with the ability to recognized RNAs have been described in human PMN, suggesting that they may become activated by the presence of RNA, the functional response of PMN to total prokaryotic RNA (pRNA) has not been previously investigated. In this sense, it has been described that of endosomal Toll-like receptors (TLRs) that bind nucleic acids, human PMN only express TLR8 (Hayashi et al., 2003; Janke et al., 2009; Berger et al., 2012). Notably, this TLR was found to be expressed and to be functional in mediating PMN effector responses when stimulated with synthetic small molecules, including imidazoquinolines (Janke et al., 2009). Moreover, it has been demonstrated that PMN express the RNA receptors, RIG-I and MDA-5, at the mRNA and protein levels (Tamassia et al., 2008; Berger et al., 2012).

Taking into account the background described above, the aim of this study was to determine the influence of bacterial viability on PMN responses and to evaluate the mechanisms involved in this phenomenon. In this regard, as the response to pRNA has been associated with microbial viability itself, we study the impact of pRNA presence on different functions of PMN.

## Materials and methods

### Ethics statement

Human normal samples were obtained from voluntary donors. This study was performed according to institutional guidelines (National Academy of Medicine, Buenos Aires, Argentina) and received the approval of the institutional ethics committee and written informed consent was provided by all the subjects.

### Blood samples

Blood samples were obtained from healthy volunteer donors who had not taken any medication for at least 10 days before the day of sampling. Blood was obtained by venipuncture of the forearm vein and was drawn directly into citrated plastic tubes.

### Polymorphonuclear neutrophil (PMN) isolation

Neutrophils were isolated by Ficoll-Hypaque gradient centrifugation (Ficoll Pharmacia, Uppsala; Hypaque, Wintthrop Products, Buenos Aires, Argentina) and dextran sedimentation, as previously described (Boyum, 1968). Contaminating erythrocytes were removed by hypotonic lysis. After washing, the cells (96% neutrophils on May Grünwald/Giemsa-stained Cyto-preps) were suspended in RPMI 1,640 supplemented with 1% heat-inactivated fetal calf serum (FCS) and used immediately after.

### Bacterial cultures and treatments

The experiments were performed using two types of gram-negative bacteria: *Escherichia coli* (ATCC® 25922™) or *Klebsiella pneumoniae* subsp. *pneumoniae* (ATCC® 700603™), and one gram-positive bacteria: *Enterococcus faecalis* (ATCC® 29212™). Different functions of PMN were evaluated against live and dead bacteria. Bacteria grown in Tryptic Soy Broth were centrifuged at 10,000 rpm for 15 min and resuspended in phosphate buffered saline (PBS) 1x. Death was induced by heat (60°C for 60 min) and irradiation (50 KGy), and was checked by the absence of bacterial growth in MacConkey agar plates for gram-negative or Tryptic Soy agar plates for gram-positive bacteria. After the corresponding treatment, the bacterial suspension was washed by centrifugation at 10,000 rpm for 15 min, the supernatant was collected to be used in some experiments, and the cell pellet was resuspended in PBS 1x. In another set of experiments, to determine if bacterial growth that occurs during the time of the experiments influence the response of PMN, the tests were performed using an auxotrophic *E. coli* strain (ZIP2286B) in PBS in the absence of FCS.

### RNA isolation

*Escherichia coli* (1 × 10^8^ CFU) or mononuclear cell (1 × 10^7^) pellets were homogenized in RNAzol-RT (MRC Gene, Cincinnati, OH) in order to obtain prokaryotic RNA (pRNA) or eukaryotic RNA (eRNA), respectively. The RNA was isolated from the resulting supernatant by alcohol precipitation, followed by washing and solubilization. An extraction control without any biological sample was also carried out in order to determine if traces of phenol and thiocyanate compounds, which could remain after the extraction of RNA, have any effect on PMN functionality (Extraction control). The concentrations of pRNA or eRNA were determined using DeNovix DS-11 Spectrophotometer (DeNovix Inc., Wilmington, DE). All samples had a 260/280 ratio between 1.8 and 2.1, and a 260/230 ratio between 1.8 and 2.3, accounting for a highly purified RNA without phenol contamination. In some experiments, pRNA was treated or not with Ribonuclease A (RNase A; Merck Millipore, Darmstadt, Germany). For total degradation, a final concentration of 5 μg/mL was used, and the samples were incubated at 37°C for 20 min. After RNase A incubation, total digestion of pRNA was checked by agarose gel electrophoresis as described below. To exclude LPS contamination in pRNA extractions, Polymyxin B (7 μg/ml; Sigma-Aldrich, MO, USA) was added to pRNA to be used as a PMN stimulus. Additionally, in some experiments, to rule out a possible role of DNA in our results, pRNA preparations were incubated with DNase I (1 U/μg of RNA; Promega Corporation, Madison, WI, USA) in a buffer containing 400 mM Tris-HCl (pH 8.0), 100 mM MgSO_4_ and 10 mM CaCl_2_ for 30 min at 37°C and the reaction was stopped by addition of 20 mM EGTA and incubation for 10 min at 65°C.

### Agarose gel electrophoresis

RNA fragments were separated via 1% agarose gel electrophoresis. 10 mg/ml of ethidium bromide was added to visualize the samples. Electrophoresis was performed at 100 V for 15 min. The RNA was detected using UV light and the size of the RNA was determined using standard 100 bp Plus DNA ladder (TransGen Biotech Co., Ltd, Beijing, China).

### Flow cytometric studies

2.5 × 10^5^ PMN were incubated with a specific mouse anti-human CD11b antibody conjugated with phycoerythrin (PE; Dako, Santa Clara, CA, USA). Debris was excluded by FSC-SSC, and the increase on FSC or CD11b expression was analyzed within the gated-viable PMN. Mean fluorescence intensity of the CD11b was determined on 50.000 events.

### Chemotaxis

Chemotaxis was quantified using a modification of the Boyden chamber technique (Betsuyaku et al., 1999). A cell suspension (50 μl) containing 2 × 10^6^ cells/ml in RPMI with 2% FCS, was placed in the top wells of a 48-well micro-chemotaxis chamber. A PVP-free polycarbonate membrane (3 μm pore size; Neuro Probe Inc. Gaithersburg MD, USA) separated the cells from lower wells containing either RPMI or the stimulus (bacteria or pRNA). The chamber was incubated for 30 min at 37°C in a 5% CO_2_ humidified atmosphere. After incubation, the filter was stained with TINCION-15 (Biopur SRL, Rosario, Argentina), and the number of PMN on the undersurface of the filter was counted in a five random high-power fields (HPF) × 400 for each of triplicate filters.

### Phagocytosis

*Escherichia coli* were conjugated with FITC (2 mg/ml) in carbonate-bicarbonate pH 9.5 1 M for 4 h at room temperature. Viability after FITC conjugation was not affected as confirmed in MacConkey agar plates (data not shown). FITC-conjugated live and HK-Ec were incubated with isolated PMN (2.5 × 10^5^ cells) for 60 min at 37°C 5% CO_2_. Immediately after, the cells were treated with Trypsin 1% in PBS 5 min at 37°C in order to remove particles deposited on the surface of the cells and finally, the cells were fixed with 0.5% paraformaldehyde and measured by flow cytometry. Alternatively, bacteria were stained with Carboxyfluorescein Diacetate Succinimidyl Ester (CFSE, Sigma-Aldrich, MO, USA) by incubating cells at 37°C and for 30 min in the dark with 10 μM of CFSE. After extensive washing, bacteria was maintained alive or heat-killed and used for phagocytosis assays.

### Reactive oxygen species (ROS) generation

To determine the production of ROS by flow cytometry DHR-123, a derivative of rhodamine 123, was used following the protocol described by Leech et al. (1998). Briefly, isolated PMN (2.5 × 10^5^) were incubated 15 min at 37°C with 1 μM DHR-123. Subsequently, the cells were incubated with or without the stimulus (bacteria or pRNA) for 30 min at 37°C 5% CO_2_ in a humidified atmosphere. Immediately after, the green fluorescence was determined.

### Neutrophil extracellular traps (NETs) formation

PMN (0.25 × 10^6^) were seeded gently onto glass coverslips coated with 0.001% poly-L-lysine in a 48-well plate in triplicate, allowed to settle, and incubated in the presence of bacteria (PMN:bacteria ratio 1:20) or RNA (10 μg/ml). Cells were incubated for 3 h at 37°C 5% CO_2_. After the incubation period, samples were gently fixed with 4% PFA, then washed with phosphate-buffered saline (PBS), and stained for DNA using Vectashield with propidium iodide (Vector Laboratories) and elastase using a specific antibody anti-PMN elastase (Merk Millipore, Darmstadt, Germany). Images for NETs evaluation were acquired using a FluoView FV1000 confocal microscope (Olympus, Tokyo, Japan) equipped with a Plapon 60x/1·42 objective lens and processed using Olympus. At least 10 different fields were observed in each triplicate (x200). NETs areas were determined in at least 5 pictures obtained in x200 using the wand tool of the FIJI software (Schindelin et al., 2012). The scale for the measurement was obtained from the data given in the confocal microscope image.

### Elastase determination

PMN (1 × 10^6^) were incubated for 3 h at 37°C 5% CO_2_ in presence of bacteria (ratio 1:20) or RNA (10 μg/ml) in RPMI medium without phenol red. After stimulation for NETs release, PMN were treated with Micrococcal Nuclease (MNasa 1 U/ml) in the presence of 1 mM CaCl_2_ for 30 min at 37°C in order to detach the NETs that were still attached to the PMN cell body. To stop MNase activity EDTA (5 mM) was added. The activity of elastase in cell-free supernatants was determined using the specific substrate N-methoxysuccinyl-ala-ala-pro-val (1 mM, Sigma-Aldrich, MO, USA) by spectrophotometry at 405 and 550 nm at 4 h post-substrate addition. As a positive control, a suspension of PMN treated with 0.5% Triton X-100 was used.

### Inhibition of endocytosis

In order to inhibit RNA endocytosis, PMN were treated for 30 min with Nystatin (30 μg/ml) prior to RNA stimulation.

### Whole blood assays

The response of PMN to pRNA was measured in whole blood with or without priming for 15 min with TNF-α (100 ng/ml). After 30 min of stimulation with pRNA, blood was treated with a lysing solution (BD, FACS Lysing Solution, San Jose, CA, USA) and the increase of CD11b expression and ROS generation were measured by flow cytometry as previously described above.

### Statistical analysis

Results were expressed as the mean ± SEM. Statistical analysis of the data was performed using the Mann-Whitney test for two group comparisons. The comparisons between multiple groups were made by analysis of variance (ANOVA), applying Tukey's post-test. *P* < 0.05 were considered significant.

## Results

### Bacterial viability is necessary to trigger PMN responses

**Figure 1:**
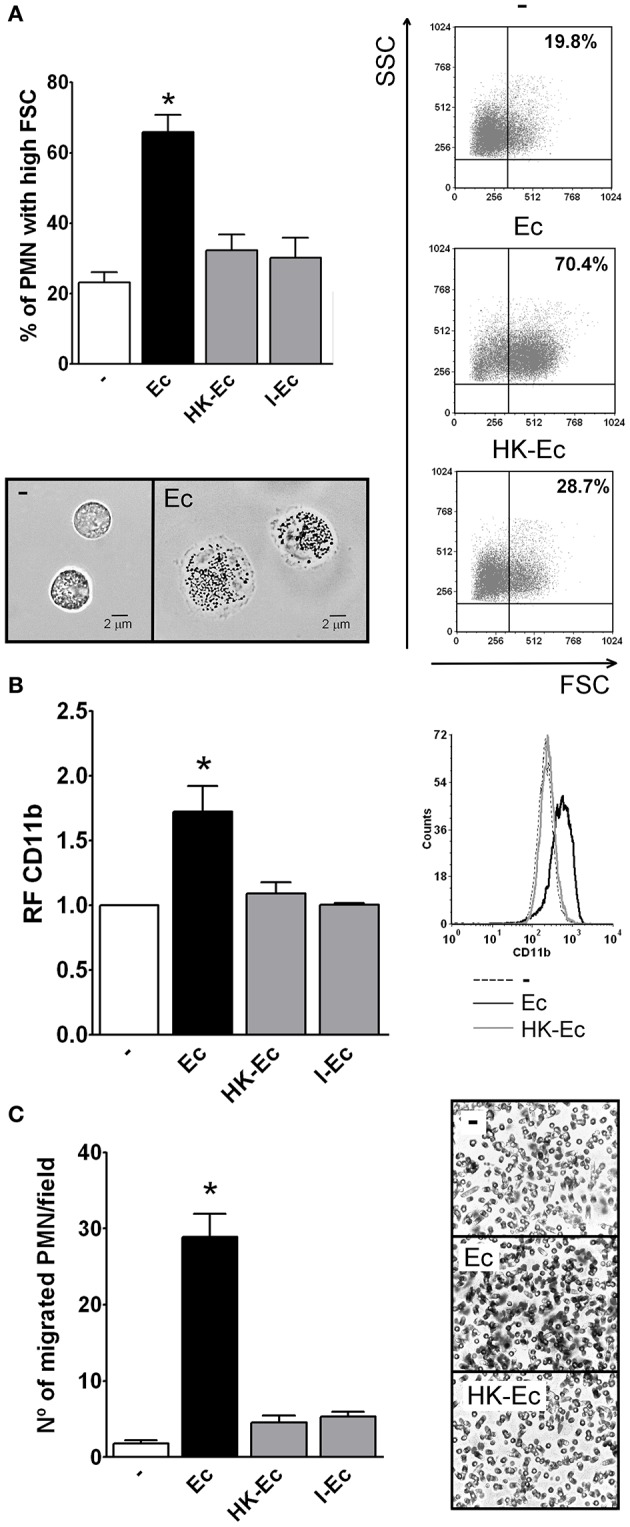
Live bacteria triggers the first steps of PMN activation. Isolated PMN were treated with live bacteria or bacteria killed by different methods in a PMN:bacteria ratio of 1:20 for 30 min. **(A)** The % of PMN that increased their FSC after stimulation with live *E. coli* (Ec), heat-killed Ec (HK-Ec), irradiated Ec (I-Ec), or left untreated (−) was determined by flow cytometry. Results were expressed as the mean ± ES, *n* = 14. *Right panel*: representative dot-plots showing SSC vs. FSC profiles of PMN with no treatment (−), Ec, and HK-Ec. *Lower panel*: Representative microphotographs of untreated PMN and PMN stimulated with Ec (x1,000). **(B)** Expression of CD11b. The mean fluorescence intensity (MFI) of the adhesion marker CD11b was determined by flow cytometry. Results were expressed as the mean ± ES of the relative fluorescence (RF) of the MFI of the different treatments with respect to untreated PMN, *n* = 8. *Right panel*: Representative histogram of the expression of CD11b in non-treated (−), Ec or HK-Ec-treated PMN. **(C)** Chemotactic response using a Boyden chamber. The number of PMN that have migrated through a 3 μm-pore membrane using Ec, HK-Ec, or I-Ec as stimulus is shown. Results were expressed as the mean ± ES, *n* = 8. *Right panel*: Representative microphotographs showing stained nuclei of PMN that migrated through the membrane with no treatment (−), Ec and HK-Ec (x1,000). In all cases ^*^p < 0.05 vs. all other groups.

Increments in the forward scatter (FSC) of PMN and CD11b up-regulation have been associated with the initial steps of spreading and activation. Therefore, we measured by flow cytometry the percentage of PMN that increased their FSC and CD11b expression after incubation with live or dead *E. coli* (Ec). The death of Ec was achieved by heating a washed bacterial suspension for 60 min at 60°C (heat-killed, HK-Ec). After this treatment, bacteria were washed, the supernatant was collected and cells were resuspended. No bacterial growth was observed in MacConkey agar plates (data not shown). As shown in Figures 1A,B, live Ec was able to increase the percentage of PMN with high FSC, and also the expression of CD11b compared to HK-Ec. In order to assess if the killing method could influence this lack of PMN responsiveness, we also used irradiated (I-Ec) bacteria, obtaining the same results as for HK-Ec.

Considering that spreading and FSC increments have been shown to correlate well with chemotaxis (Cole et al., 1995), we used a Boyden chamber to determine the ability of Ec or HK-Ec to function as a chemotactic stimulus. Figure 1C shows that only live Ec was able to induce PMN migration compared to HK-Ec or Ec killed by other methods.

As depicted in the (Figure S1), live Ec stimulates PMN functions in a dose-dependent manner and dead bacteria showed weak or no stimulation up to ratios of PMN:bacteria 1:100. When higher ratios were used, although dead bacteria showed some effects on PMN, equivalent levels of live bacteria induced toxic effects.

**Figure 2:**
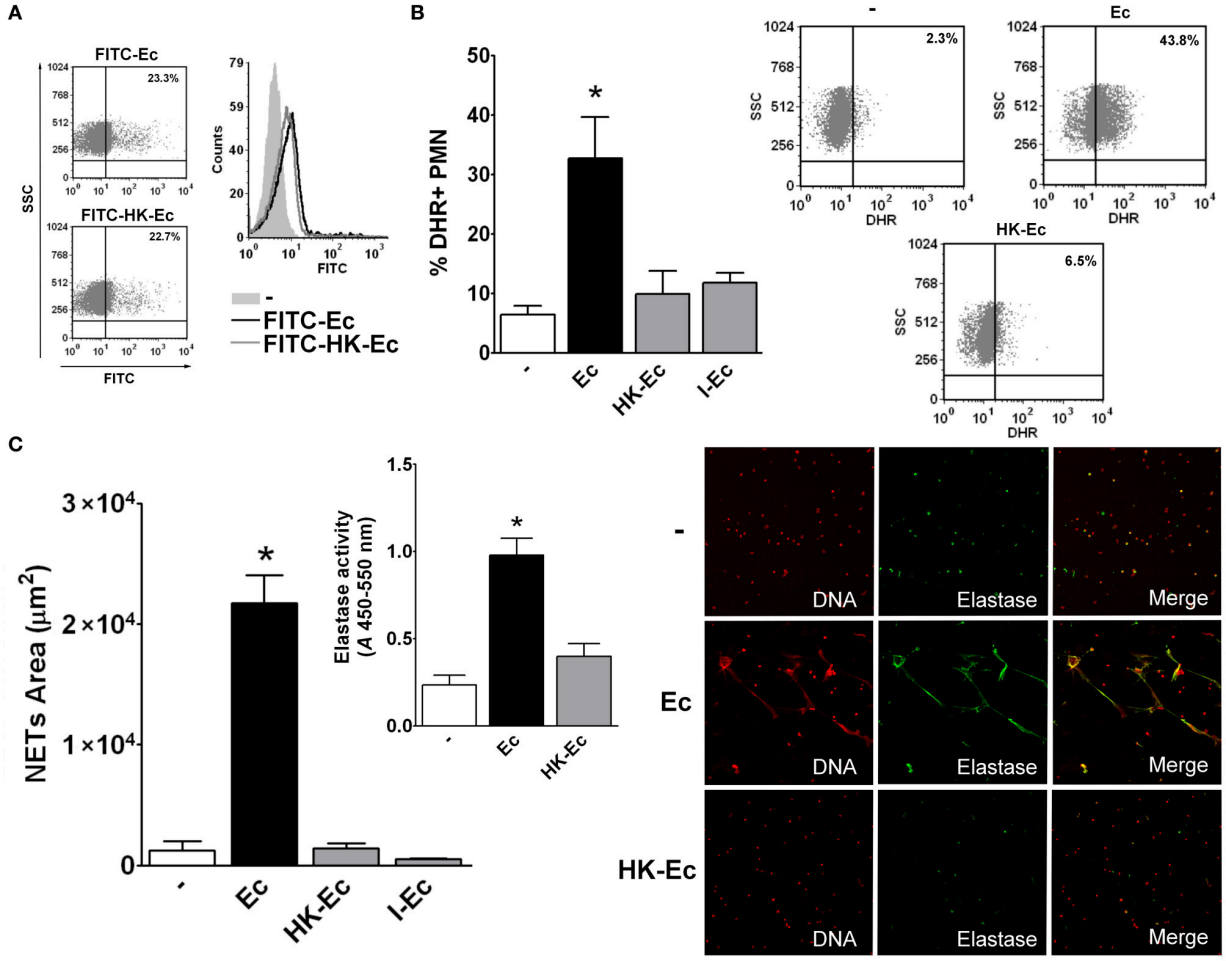
Live bacteria is critical for triggering bactericidal responses. **(A)** Phagocytosis of FITC-Ec or FITC-HK-Ec was determined after 60 min by flow cytometry. Dot plots showing the percentage and a histogram showing the MFI of FITC+ PMN of a representative experiment out of three are depicted. **(B)** ROS generation. Isolated PMN were stained with DHR for 15 min and then treated with Ec, HK-Ec, or I-Ec in a PMN:bacteria ratio of 1:20 for 30 min. The % of DHR+ PMN was determined by flow cytometry. Results were expressed as the mean ± ES, *n* = 8. *Right panel*: Representative dot-plots of the percentage of DHR+ PMN are shown in non-treated (−), Ec and HK-Ec-treated PMN. **(C)** Isolated PMN were incubated with Ec, HK-Ec, or I-Ec in a PMN:bacteria ratio of 1:20 for 3 h and NETs were stained with propidium iodide for DNA and with a specific antibody for elastase detection and were visualized by confocal microscopy. NETs area (μm^2^) was measured using the FIJI software as described in material and methods. Results were expressed as the mean ± ES, *n* = 10. *Inset:* Elastase activity using a specific substrate was determined and the absorbance (*A*) at 450–550 nm is depicted for untreated PMN (−) and PMN treated with Ec or HK-Ec. Results were expressed as the mean ± ES, *n* = 10. *Right panel*: Representative microphotographs of confocal images showing PMN from the untreated (−), Ec and HK-Ec groups stained for DNA or elastase visualization and the merge of the images (x200). In all cases ^*^p < 0.05 vs. all other groups.

Given the above results, at this point, it was important to determine if HK-Ec and live Ec were equally phagocyted. Therefore, we used Ec conjugated with FITC and evaluated by flow cytometry phagocytosis of live and HK-Ec. As shown in Figure 2A, both the percentage and the amount of bacteria internalized per cell (MFI) were similar for live and HK-Ec. The same results were obtained when bacteria were stained with CSFE (data not shown).

We then examine two PMN functions that are more associated with their bactericidal ability: ROS generation and NETs formation. As Figures 2B,C depicts, both ROS and NETs were triggered only when Ec was alive and were not induced by HK-Ec or I-Ec.

As it was possible that the differences observed with live or dead bacteria could be ascribed to bacterial growth (in live Ec) during the time in which the assays were performed (especially considering the 3-h incubation for NETs visualization), we used an auxotrophic Ec (Aux-Ec), and repeated all the experiments in PBS 1x in the absence of FCS. In these conditions (PBS 1x, no FCS), live Aux-Ec did not increase their CFU after 30 min or 3 h at 37°C and 5% CO_2_ as assessed in McConkey agar plates (CFU Time 0 = 5.2 × 10^6^, 30 min = 4.8 × 10^6^, 3 h= 5.5 × 10^6^). Therefore, the use of Aux-Ec ensures that the initial bacterial number is the same at the end of the assay for live and dead Ec. As Figure S2 depicts, the differences between live and HK-Aux-Ec were statistically significant for all the assays previously performed, and the responses using live Aux-Ec were similar to those observed with live wild-type Ec.

Moreover, as shown in Figure S3, viability requirement for inducing PMN activation was not exclusive of Ec, as we also observed the same differences in the induction of PMN activation between live and HK bacteria for the Gram-negative *K. pneumoniae* (Kp), and the Gram-positive *E. faecalis* (Ef) strains.

Altogether, our results show that live and not dead bacteria are able to induce a strong PMN response, suggesting that bacterial determinants associated with viability are necessary to trigger PMN activation.

### Heat killing leads to loss of RNA content in EC

**Figure 3:**
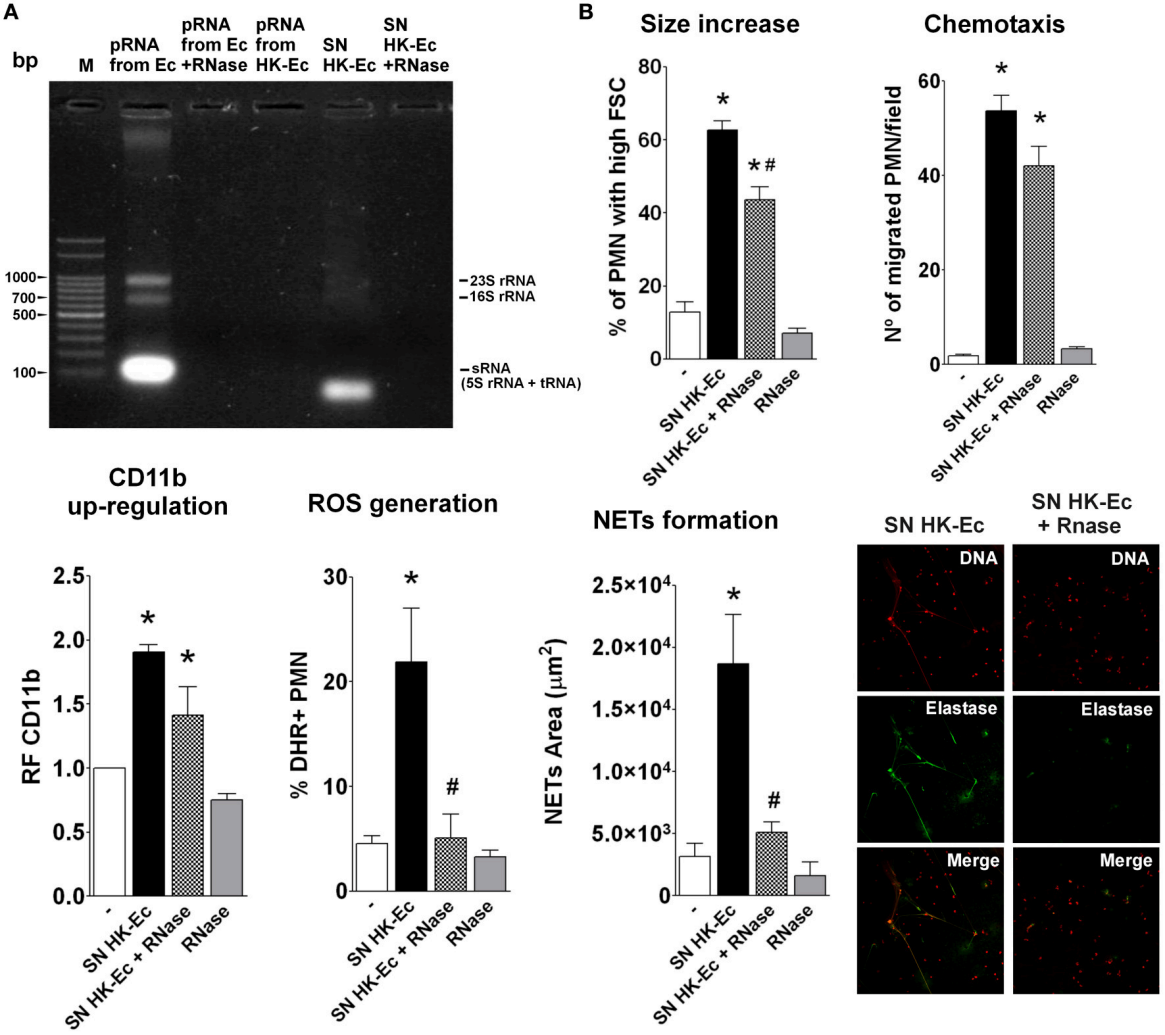
Death caused the release of RNA from bacteria. **(A)** Agarose gel electrophoresis stained with ethidium bromide. M-Marker: 100 bp Plus DNA ladder. **(B)** PMN activation was determined using the bacteria-free supernatants (SN) of HK-Ec, the SN treated with RNase (SN HK-Ec+RNase) and RNase alone by measuring different functions as detailed for Figures 1, 2. Results were expressed as the mean ± ES, *n* = 5. ^*^p < 0.05 vs. non-treated PMN (−), ^#^*p* < 0.05 vs. SN HK-Ec.

Considering that prokaryotic RNA (pRNA) has been reported as a PAMP associated to bacterial viability (Sander et al., 2012), we asked if there was any difference at the RNA level between live and dead bacteria. For this purpose, we extracted RNA from live and dead bacteria. As Figure 3A depicts, RNA extractions from live Ec (pRNA from Ec) showed the three typical band pattern in an agarose gel electrophoresis, that disappeared after RNase A digestion (pRNA form Ec + RNase). Surprisingly, RNA could not be isolated from HK-Ec using a standard protocol of RNA extraction, as assessed by gel agarose electrophoresis and by the absence of any detectable concentration of RNA measured at 260 nm. The supernatant of HK-Ec (SN HK-Ec) shows a band of lower molecular weight that was completely lost by RNase treatment (SN HK-Ec + RNase). These results indicate that death of bacteria by heat treatment induces the release of RNA to the extracellular milieu, suggesting that bacterial viability-induced PMN activation may be associated with the presence of RNA in live bacteria.

Accordingly, when we assayed these bacteria-free supernatants as stimuli for PMN activation, we found that they were able to induce an up-regulation of CD11b, increases in cell size, chemotaxis, ROS, and NETs generation (Figure 3B). RNase treatment of these supernatants was able to completely abolish the effects of the supernatants on ROS and NETs, partially decreased size increase and had no influence on chemotaxis and CD11b up-regulation. These results indicate that the pRNA released after bacterial death is crucial in triggering bactericidal functions of PMN. Moreover, as initial activation of PMN is still observed after RNAse treatment, it is possible that other factors released in the supernatants or RNase degradation products could be mediating these effects.

As depicted in Figure S4, the supernatants obtained from live Ec were also able to stimulate PMN functions. However, no pRNA could be detected in these supernatants in agarose gel electrophoresis. Moreover, pRNA was also not detected after a protocol of RNA extraction of these supernatants, and these preparations were unable to stimulate any of the PMN responses assayed. These results indicate that replicating bacteria is also able to release mediators, different from RNA, that activate PMN. However, these PMN-activating mediators are not purified in the process of RNA isolation.

### pRNA is able to directly activate PMN responses

**Figure 4:**
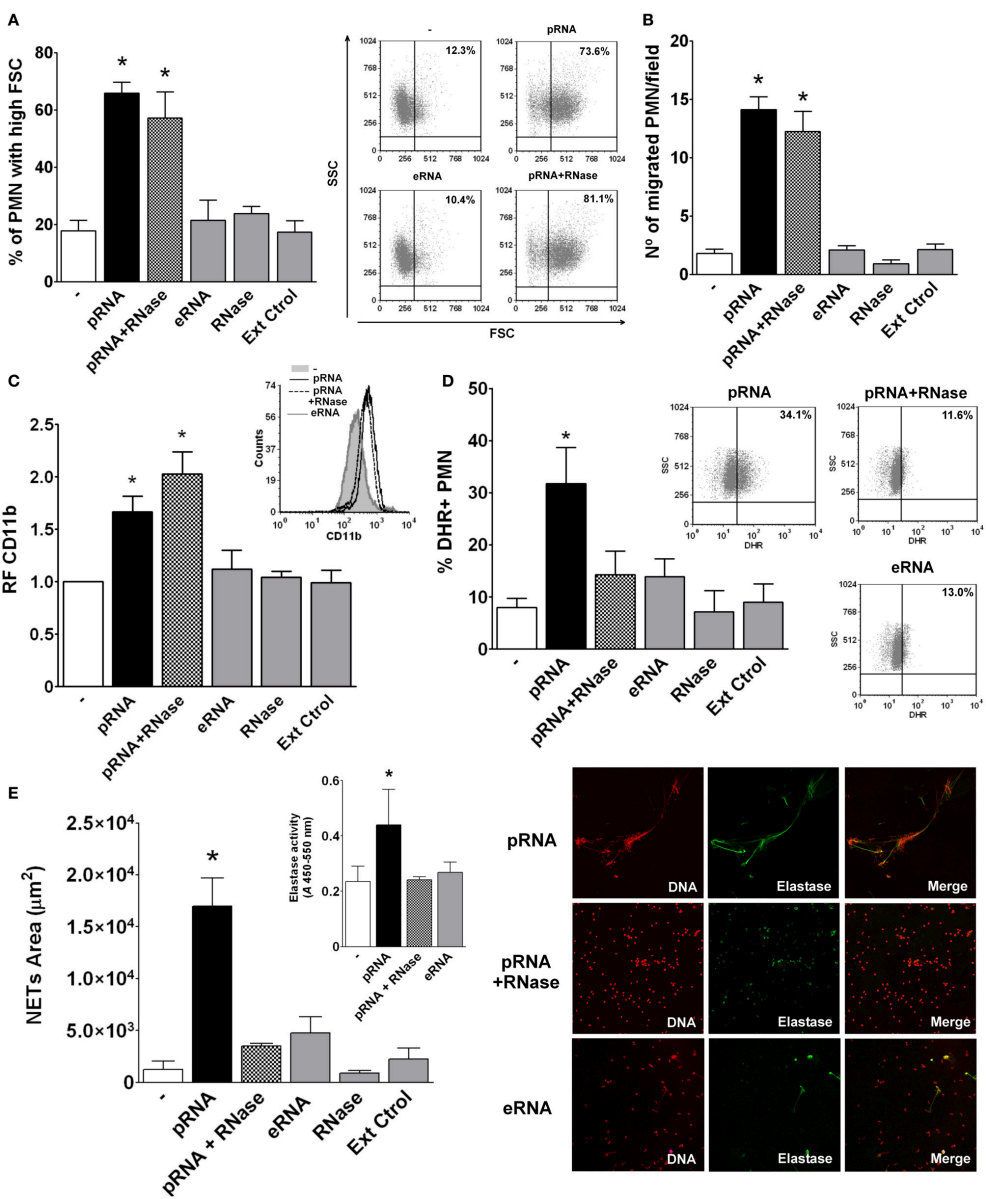
Prokaryotic RNA (pRNA) directly activated PMN. Isolated PMN were treated with purified pRNA, eukaryotic RNA (eRNA), or pRNA treated with RNase. Controls of RNase alone or an extraction control (Ext Ctrol), using water instead of bacteria, were also included. **(A)** Percentage of PMN with high FSC. Results were expressed as the mean ± ES, *n* = 15. *Right panel*: representative dot-plots showing SSC vs. FSC profiles of PMN with no treatment (−), pRNA, eRNA, or pRNA+RNase. **(B)** Chemotactic response. Results were expressed as the mean ± ES, *n* = 7. **(C)** Expression of CD11b. Results were expressed as the mean ± ES of the relative fluorescence (RF) of the MFI of the different treatments with respect to untreated PMN, *n* = 7. *Right panel*: Representative histogram of the expression of CD11b in untreated PMN (−), or PMN incubated with pRNA, eRNA, and pRNA+RNase. **(D)** ROS generation. Results were expressed as the mean ± ES, *n* = 8. *Right panel*: Representative dot-plots of the percentage of DHR+ PMN incubated with pRNA, pRNA+RNase, or eRNA. **(E)** NETs formation. Results were expressed as the mean ± ES, *n* = 9. *Inset:* Elastase activity for untreated PMN (-), or pRNA, pRNA+RNase, and eRNA treatments. Results were expressed as the mean ± ES, *n* = 9. *Right panel*: Representative microphotographs of confocal images showing PMN from pRNA, pRNA+RNase, and eRNA groups stained for DNA or elastase visualization and the merge of the images (x200). In all cases ^*^p < 0.05 vs. all other groups.

According to the above results and in order to study directly the effects of pRNA on PMN activation, we isolated pRNA from Ec and used it as a stimulus. Similarly to live Ec, pRNA induced an increase in FSC, chemotaxis, CD11b expression, ROS generation and NETs formation (Figures 4A–E). Moreover, PMN activation by pRNA was dose-dependent as shown in Figure S5. Moreover, the contribution of LPS or DNA as possible contaminating substances of our pRNA preparations in the observed PMN activation was analyzed by incubation of PMN with pRNA, pRNA+polymyxin B, or pRNA+DNase. We found that the presence of polymyxin B or DNA degradation did not alter the increases in FSC, CD11b expression and ROS production induced by pRNA, ruling out the possibility that contaminating LPS or DNA in our pRNA preparations could be mediating PMN activation (Figure S6).

To further characterize pRNA-induced effects, we first asked if the phenomenon was exclusive for RNA of a prokaryotic origin. In this sense, RNA extracted from eukaryotic human cells (eRNA) was unable to trigger PMN activation (Figures 4A–E). Moreover, and in concordance to what we observed in the supernatant of HK-Ec, degradation of pRNA by RNase abolished the pRNA effects in ROS and NETs, but it was still able to induce an increase in FSC, CD11b up-regulation, and chemotaxis. These results indicate that pRNA, but not eRNA, is a direct activating stimulus for PMN. Additionally, pRNA, even when degraded, may stimulate PMN functions implicated in movement and degranulation, while other functions involved in bactericidal mechanisms need more conserved RNA molecules.

### Mechanisms involved in pRNA-induced PMN activation

**Figure 5:**
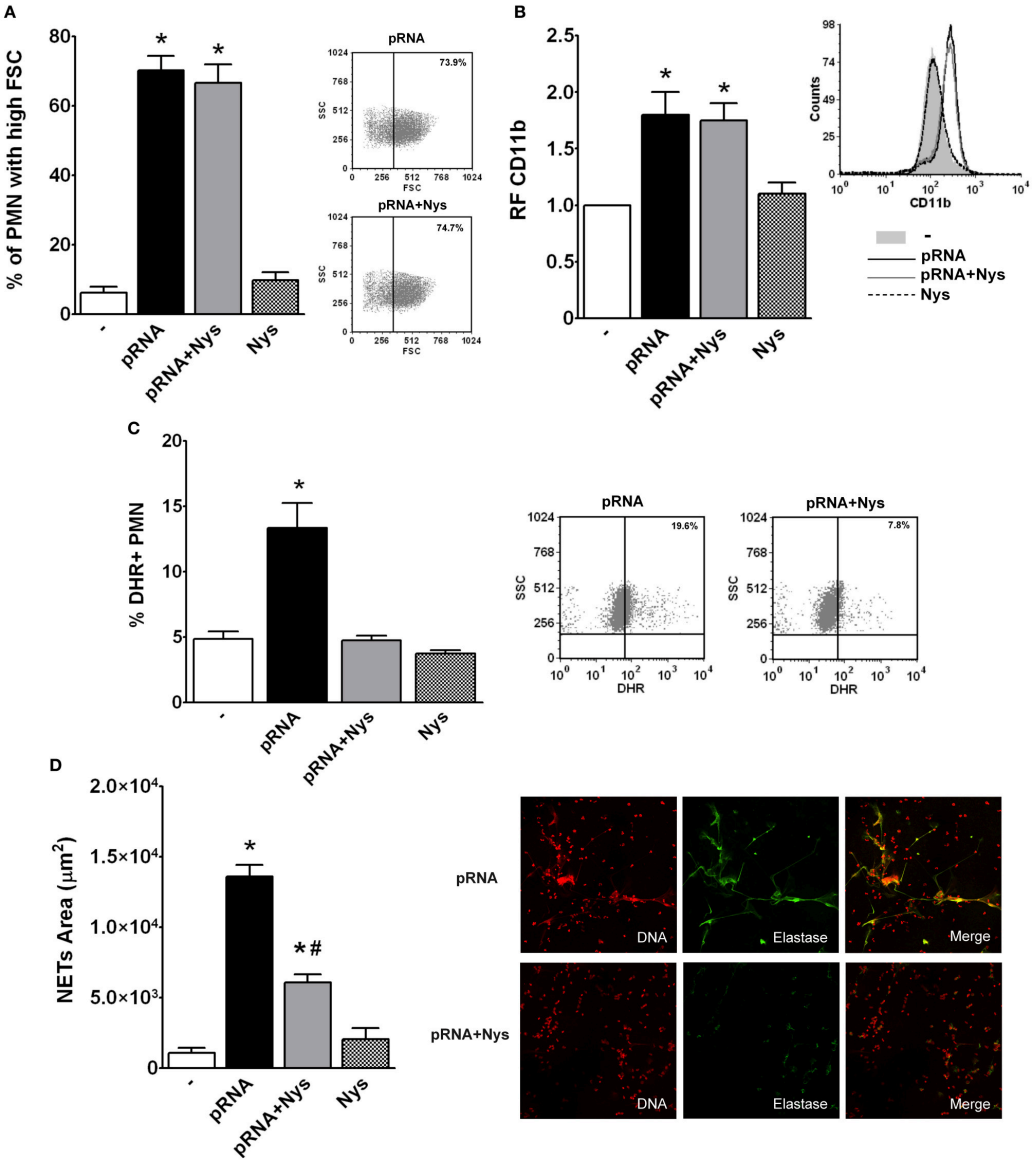
Endocytosis of pRNA is needed for triggering bactericidal responses. Isolated PMN were incubated with pRNA in the presence or absence of the endocytosis inhibitor, Nystatin (Nys). Results were expressed as the mean ± ES, *n* = 5. **(A)** Percentage of FSC high PMN. *Right panel*: Representative dot-plots showing the SSC vs. FSC profiles of PMN incubated with pRNA or pRNA+Nys. **(B)** Expression of CD11b. Results were expressed as the relative fluorescence (RF) of the MFI of the different treatments with respect to untreated PMN. *Right panel*: Representative histogram showing the CD11b expression of PMN left untreated or incubated with pRNA, pRNA+Nys, and Nys. **(C)** ROS generation. *Right panel*: Representative dot-plots of the percentage of DHR+ PMN are shown in untreated (−) or incubated with pRNA, pRNA+Nys, and Nys. **(D)** NETs formation. *Right panel*: Representative microphotographs of confocal images showing PMN from pRNA and pRNA+Nys groups stained for DNA or elastase visualization and the merge of the images (x200). In all cases ^*^p < 0.05 vs. all other groups and ^#^*p* < 0.05 vs. pRNA.

As RNA recognition receptors are usually intracellular, we then asked whether internalization of pRNA is necessary to trigger PMN responses. For this purpose, we used Nystatin, a cholesterol-sequestering antifungal agent that inhibits endocytosis (Rothberg et al., 1992; Puri et al., 2001). PMN were treated with Nystatin (Nys) and then incubated with pRNA (pRNA+Nys). As shown in Figure 5, Nystatin treatment inhibited pRNA-induced ROS and NETs generation, without affecting, neither the increase in the % of PMN with high FSC or up-regulation of CD11b.

**Figure 6:**
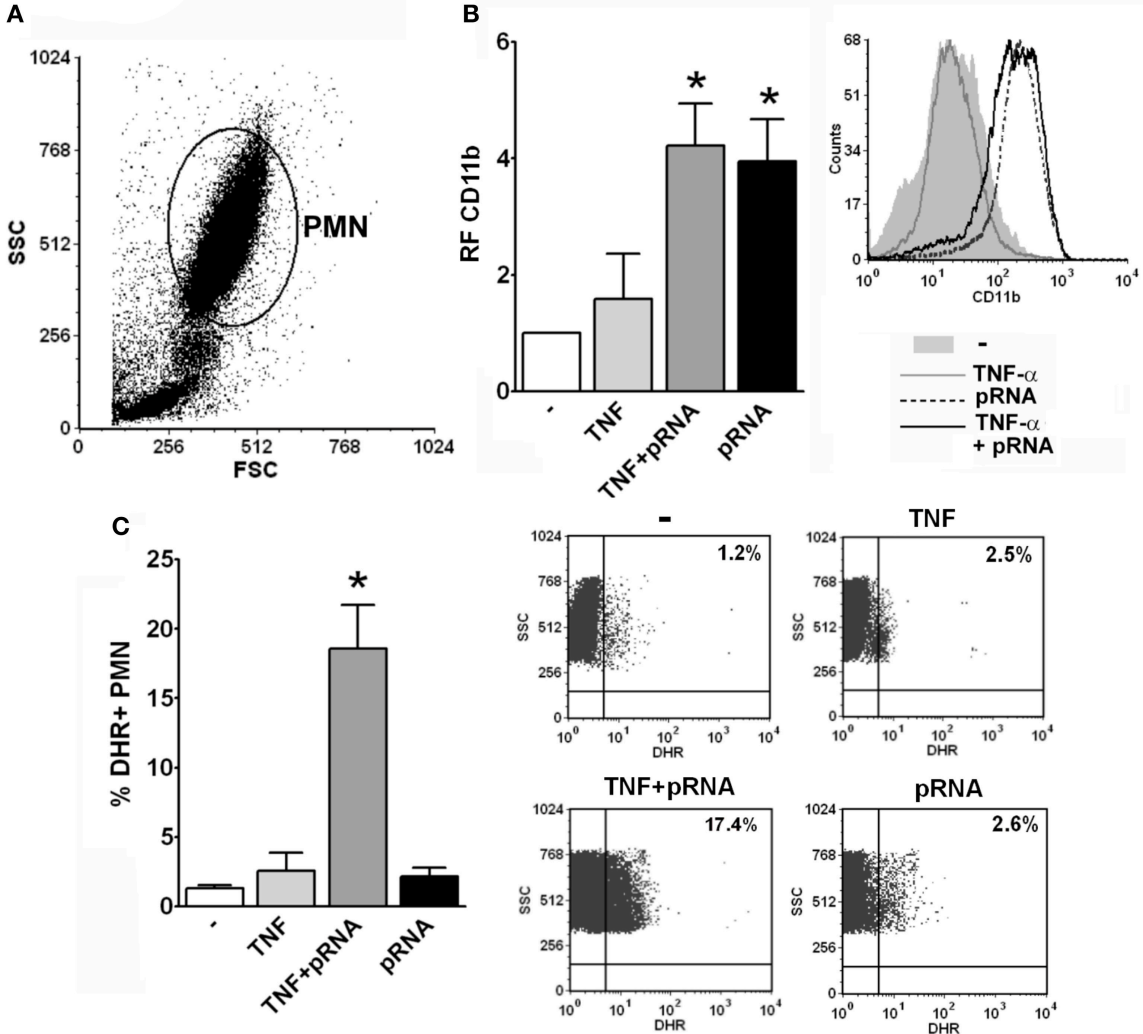
pRNA-induced ROS requires priming. **(A)** Representative dot-plot showing the SSC vs. FSC profile of the different populations observed in whole blood assays after lysis of erythrocytes using a commercial lysing buffer. The PMN population is indicated. **(B)** Whole blood was incubated with pRNA and/or the priming stimulus, TNF-α, for 30 min and the expression of CD11b was measured by flow cytometry within the gate of PMN. A histogram of one representative experiment out of three is shown. **(C)** Whole blood was incubated with DHR for 15 min and with the different stimuli for additional 30 min. The % of DHR+ PMN was determined by flow cytometry within the gate of PMN. Dot-plots of the percentage of DHR+ PMN of one representative experiment out of three are shown. In all cases ^*^p < 0.05 vs. all other groups.

As the purification process may lead to PMN priming, we measured the response of PMN to pRNA in whole blood with or without priming with TNF-α in order to investigate if RNA effects on PMN also occur in resting PMN or require a priming mechanism. Unfortunately, for methodological reasons, not all the assays measured in purified PMN are able to be performed in whole blood. Nevertheless, as depicted in Figure 6, we could measure CD11b expression and ROS generation and found that up-regulation of CD11b by pRNA was triggered in whole blood without previous PMN priming. However, ROS generation was induced by pRNA only when PMN were previously incubated with the priming stimulus. These results indicate that endocytosis of pRNA and priming of PMN are necessary for triggering some functions but not others.

## Discussion

Initially, the process of PMN activation triggers a cascade of events that lead to spreading. After that, interaction with endothelial cells by up-regulation of integrins, such as CD11b, occurs to undergo migration through the endothelium. Upon bacterial encounter, PMN killed bacteria either intracellularly after phagocytosis and ROS generation or extracellularly by NETs formation. In this work, we first evaluate the necessity of bacterial viability to modulate PMN functionality, measuring parameters associated with different steps of PMN activation. Our results indicate that from the initial increase in FSC until the activation of bactericidal mechanisms, in PMN:bacteria ratios compatible with infection, and not intoxication, PMN can only respond when challenged with live bacteria and we also demonstrate that this phenomenon is not exclusive for *E. coli*, and may be extended to other bacterial strains.

The fact that PMN only responds to live bacterial stimulation is probably associated with the loss of pRNA in dead bacteria and its release into the extracellular milieu. In this sense, no RNA could be isolated from HK-Ec, and a band corresponding to RNA was observed in the supernatants of HK-Ec in agarose gel electrophoresis. This band showed a lower molecular weight, probably corresponding to partially degraded products. In this sense, it is possible that the heat killing process induces intracellular degradation of RNA and the release of fragmented RNA. The absence of RNA in the supernatant of live Ec reinforces the fact that pRNA is release as a consequence of bacterial death. The release of RNA into the extracellular milieu may be compatible with bacteria dying by extracellular mechanisms, such as NETosis. Therefore, the addition of pRNA extracellularly in our experimental conditions represents a physiological possible scenario in an infectious context. RNase treatment of HK-Ec supernatants revealed that non-degraded RNA molecules were needed for triggering ROS and NETs generation, although the presence of other molecules, and even degraded RNA, may be also triggering other PMN functions.

Although several molecules released from live or dead bacteria could stimulate PMN, we found that isolated total pRNA mimicked PMN activation by live bacteria. For ensuring that the observed PMN activation was mediated by pRNA, we performed several controls. We have always used preparations of isolated RNA with a high 260/280 ratio (>1.8); we have included extractions controls to exclude the influence of phenolic components of RNAzol that would persist after isolation; we have performed experiments in the presence of polymyxin B to exclude the contribution of possible contaminating lipopolysaccharide, and also treated pRNA preparations with DNAse to rule out the effect of possible contaminating DNA. Taking into account the results obtained with all these controls, we believe that pRNA is the molecule triggering all the functions assayed, especially when considering that it is unlikely that another bacterial component, not present in dead bacteria, could be co-purified with pRNA in a relevant proportion to trigger PMN activation. In line with this, although the supernatant of live Ec (devoid of RNA) contained other molecules that stimulated PMN, after the RNA extraction protocol these supernatants lost their PMN-stimulating activity, reinforcing the fact that the RNA extraction protocol does not co-purify other PMN-stimulating molecules. In this sense, as increase in cell size, CD11b up-regulation and chemotaxis were also triggered when pRNA was digested by RNase treatment, and in the absence of other PMN-activating mediators in RNA preparations, degraded pRNA may be also triggering some PMN functions, although the mechanism involved in this phenomenon needs further investigation.

When we studied the mechanisms required for triggering activation by pRNA, we found that ROS and NETs formation were abolished when pRNA was degraded, and were dependent on endocytosis. PMN have been shown to express some receptors that can recognize RNA, and may potentially be involved in the activation triggered by pRNA in PMN. In this sense, from all TLRs responsible for RNA recognition, PMN only express endosomal TLR8 (Hayashi et al., 2003; Janke et al., 2009; Berger et al., 2012). Another possible intracellular receptor could be NLRP3, a cytosolic component of the inflammasome present and activable in PMN (Bakele et al., 2014; Pérez-Figueroa et al., 2016). In this sense, Sander et al. have demonstrated that RNA from viable bacteria could gain access to cytosolic receptors via intrinsic phagosomal leakage reaching the NLRP3 inflammasome (Sander et al., 2012). Regardless of the receptor involved, RNA internalization would be a requirement for interaction with any of these receptors. In line with this, in our experimental settings, the inhibition of endocytosis abolished pRNA-induced ROS and NETs formation and indicates, although indirectly, that pRNA is internalized by PMN. Another RNA receptor present in PMN is RIG-I. Interestingly, although this receptor was regarded as an intracellular cytosolic RNA receptor for other cell types, in resting (not primed) PMN RIG-I was found, besides the cytoplasm, in secretory vesicles and at the cell surface, suggesting that pRNA recognition in PMN could also occur at the plasmatic membrane level as well (Berger et al., 2012). The interaction of purified pRNA with RIG-I at the plasmatic membrane could explain the induction of PMN functions that were also triggered in the presence of the endocytosis inhibitor, although this needs further investigation.

Moreover, priming was also necessary to trigger ROS by pRNA. Although the need for priming could raise doubts about the validity of the results found in purified PMN, in an infectious context PMN will surely be primed, as the presence of pRNA will always be accompanied by priming mediators, such as bacterial structural components or pro-inflammatory cytokines.

Our results demonstrate, for the first time, the role of pRNA as an inducer of PMN activation. In an infectious focus, chemoattractant bacterial compounds will attract PMN and will favor adhesion of these cells to endothelium. Once at the site of infection, bactericidal mechanisms will be triggered by phagocyted RNA-carrying live bacteria. ROS, intracellularly, and NETs, extracellularly, will begin to control the infection. The release of pRNA by extracellularly, NETs-induced, dying bacteria will create an amplification loop that will call more PMN to the site of infection.

In summary, our results shed light in understanding how PMN actively gauge the infectious risk by recognizing signatures of microbial life and thus infectivity. Moreover, our work shows the important role of bacteria-derived exogenous RNA in host-microbe interactions and may help to elucidate new mechanisms associated with pathogenesis and, perhaps, allow the identification of new drug targets.

## Author contributions

NR, VL, PB, and GF conceived and designed the experiments. NR, LC, DM, and MM performed the experiments. NR, VL, and GF analyzed and interpreted the data. PB and MM contributed with reagents/materials/analysis tools. GF and NR wrote the manuscript. VL and PB revised the manuscript. NR, LC, VL, DM, MM, PB, and GF approved the version to be published, and agreed to be accountable for all aspects of the work in ensuring that questions related to the accuracy or integrity of any part of the work are appropriately investigated and resolved.

### Conflict of interest statement

The authors declare that the research was conducted in the absence of any commercial or financial relationships that could be construed as a potential conflict of interest.

## Abbreviations

PMN: Polymorphonuclear neutrophils
PAMP: Pathogen-associated molecular patterns
PRP: Pattern recognition receptors
TLR: Toll-like receptor
FSC: Forward scatter
SSC: Side scatter
FITC: Fluorescein isothiocyanate
MFI: Mean fluorescence intensity
DHR: Dihydrorhodamine 123
ROS: Reactive oxygen species
NET: Neutrophil extracellular traps
FCS: fetal calf serum
Ec: *Escherichia coli*
Kp: *Klebsiella pneumoniae*
Ef: *Enterococcus faecalis*
HK: Heat-Killed
I: Irradiated
Aux: Auxotrophic
SN: Supernatant
pRNA: prokaryotic ribonucleic acid
eRNA: eukaryotic ribonucleic acid
RNase: Ribonuclease
Nys: Nystatin.

## References

[B1] Amulic B. Hayes G. (2011). Neutrophil extracellular traps. Curr. Biol. 21, R297–R298. 10.1016/j.cub.2011.03.021 21549944

[B2] Bakele M. Joos M. Burdi S. Allgaier N. Pöschel S. Fehrenbacher B.. (2014). Localization and functionality of the inflammasome in neutrophils. J. Biol. Chem. 289, 5320–5329. 10.1074/jbc.M113.505636 24398679 PMC3931087

[B3] Berger M. Hsieh C. Y. Bakele M. Marcos V. Rieber N. Kormann M.. (2012). Neutrophils express distinct RNA receptors in a non-canonical way. J. Biol. Chem. 287, 19409–19417. 10.1074/jbc.M112.353557 22532562 PMC3365979

[B4] Betsuyaku T. Liu F. Senior R. M. Haug J. S. Brown E. J. Jones S. L.. (1999). A functional granulocyte colony-stimulating factor receptor is required for normal chemoattractant-induced neutrophil activation. J. Clin. Invest. 103, 825–832. 10.1172/JCI5191 10079103 PMC408143

[B5] Boyum A. (1968). Separation of leukocytes from blood and bone marrow. Introduction. Scand. J. Clin. Lab. Invest. Suppl. 97, 7. 5707208

[B6] Cole A. T. Garlick N. M. Galvin A. M. Hawkey C. J. Robins R. A. (1995). A flow cytometric method to measure shape change of human neutrophils. Clin. Sci. 89, 549–554. 10.1042/cs0890549 8549071

[B7] Detmer A. Glenting J. (2006). Live bacterial vaccines–a review and identification of potential hazards. Microb. Cell Fact. 5:23. 10.1186/1475-2859-5-23 16796731 PMC1538998

[B8] El-Benna J. Hurtado-Nedelec M. Marzaioli V. Marie J. C. Gougerot-Pocidalo M. A. Dang P. M. (2016). Priming of the neutrophil respiratory burst: role in host defense and inflammation. Immunol. Rev. 273, 180–193. 10.1111/imr.12447 27558335

[B9] Hayashi F. Means T. K. Luster A. D. (2003). Toll-like receptors stimulate human neutrophil function. Blood 102, 2660–2669. 10.1182/blood-2003-04-1078 12829592

[B10] Janke M. Poth J. Wimmenauer V. Giese T. Coch C. Barchet W.. (2009). Selective and direct activation of human neutrophils but not eosinophils by Toll-like receptor 8. J. Allergy Clin. Immunol. 123, 1026–1033. 10.1016/j.jaci.2009.02.015 19361845

[B11] Lacy P. (2006). Mechanisms of degranulation in neutrophils. Allergy Asthma Clin. Immunol. 2, 98–108. 10.1186/1710-1492-2-3-98 20525154 PMC2876182

[B12] Leech M. Hutchinson P. Holdsworth S. R. Morand E. F. (1998). Endogenous glucocorticoids modulate neutrophil migration and synovial P-selectin but not neutrophil phagocytic or oxidative function in experimental arthritis. Clin. Exp. Immunol. 112, 383–388. 10.1046/j.1365-2249.1998.00601.x 9649205 PMC1905001

[B13] McDonald B. Pittman K. Menezes G. B. Hirota S. A. Slaba I. Waterhouse C. C.. (2010). Intravascular danger signals guide neutrophils to sites of sterile inflammation. Science 330, 362–366. 10.1126/science.1195491 20947763

[B14] Pérez-Figueroa E. Torres J. Sánchez-Zauco N. Contreras-Ramos A. Alvarez-Arellano L. Maldonado-Bernal C. (2016). Activation of NLRP3 inflammasome in human neutrophils by *Helicobacter pylori* infection. Innate Immun. 22, 103–112. 10.1177/1753425915619475 26610398

[B15] Puri V. Watanabe R. Singh R. D. Dominguez M. Brown J. C. Wheatley C. L.. (2001). Clathrin-dependent and -independent internalization of plasma membrane sphingolipids initiates two Golgi targeting pathways. J. Cell Biol. 154, 535–547. 10.1083/jcb.200102084 11481344 PMC2196434

[B16] Rothberg K. G. Heuser J. E. Donzell W. C. Ying Y. S. Glenney J. R. Anderson R. G. (1992). Caveolin, a protein component of caveolae membrane coats. Cell 68, 673–682. 10.1016/0092-8674(92)90143-Z 1739974

[B17] Sander L. E. Davis M. J. Boekschoten M. V. Amsen D. Dascher C. C. Ryffel B.. (2012). Detection of prokaryotic mRNA signifies microbial viability and promotes immunity. Nature 474, 385–389. 10.1038/nature10072 21602824 PMC3289942

[B18] Schindelin J. Arganda-Carreras I. Frise E. Kaynig V. Longair M. Pietzsch T.. (2012). Fiji: an open-source platform for biological-image analysis. Nat. Methods 9, 676–682. 10.1038/nmeth.2019 22743772 PMC3855844

[B19] Tamassia N. Le Moigne V. Rossato M. Donini M. McCartney S. Calzetti F.. (2008). Activation of an immunoregulatory and antiviral gene expression program in poly(I:C)-transfected human neutrophils. J. Immunol. 181, 6563–6573. 10.4049/jimmunol.181.9.6563 18941247

