# Supplementary figures and images for: Prokaryotic RNA Associated to Bacterial Viability Induces Polymorphonuclear Neutrophil Activation
Source: Front Cell Infect Microbiol. 2017 Jul 6;7:306. doi: 10.3389/fcimb.2017.00306 (PMC5498479; doi:10.3389/fcimb.2017.00306)

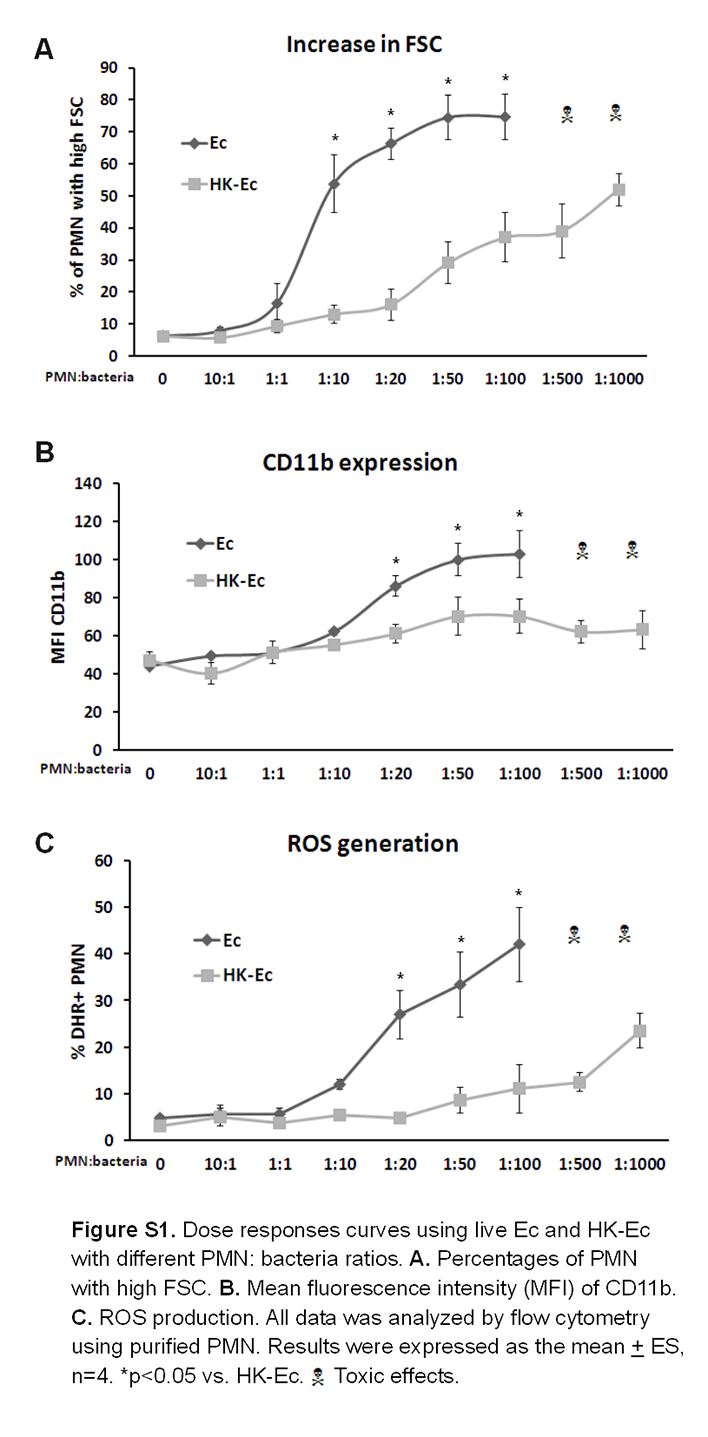

Supplement: Supplementary file 1 [file Image1.TIF]

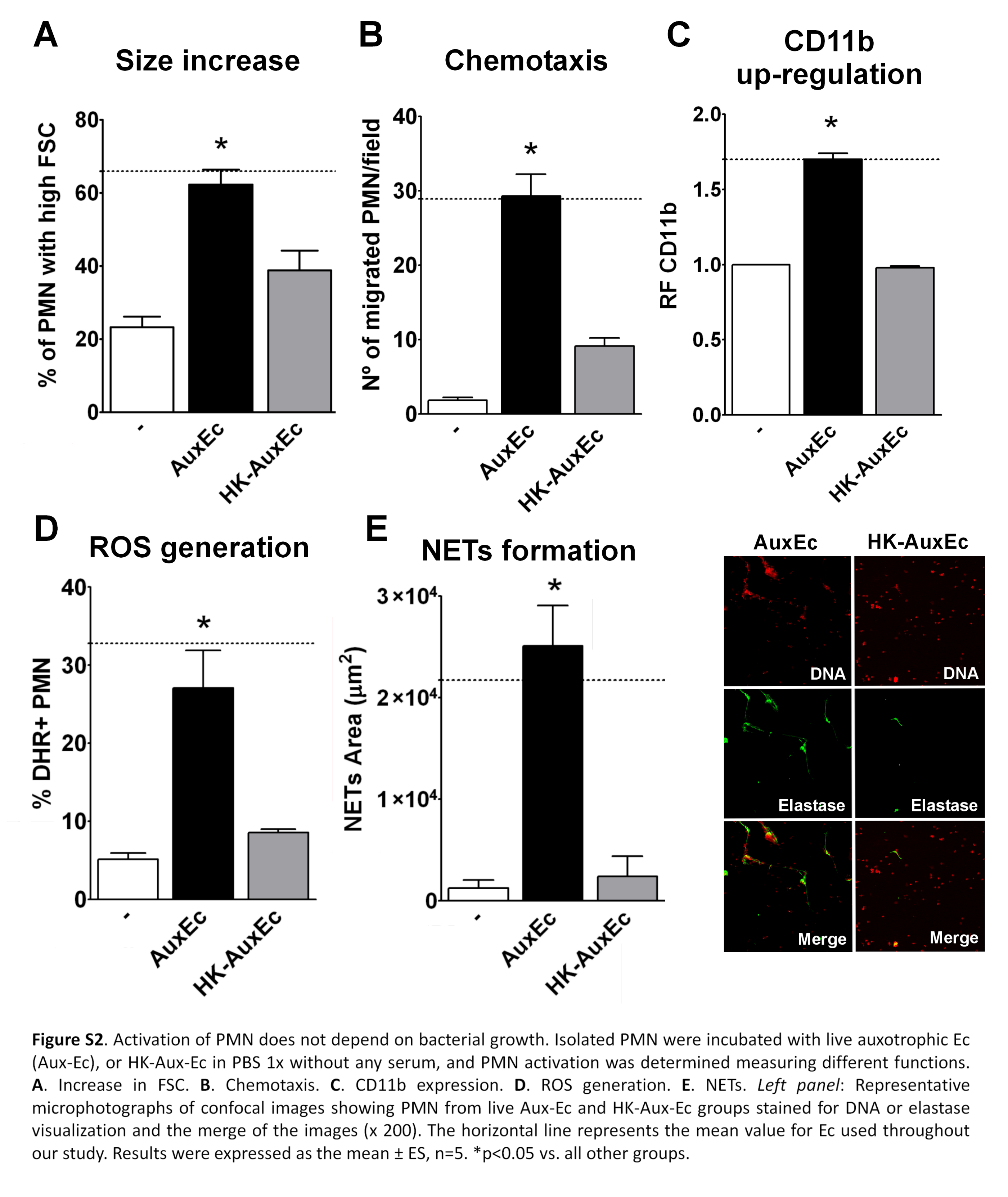

Supplement: Supplementary file 2 [file Image2.TIF]

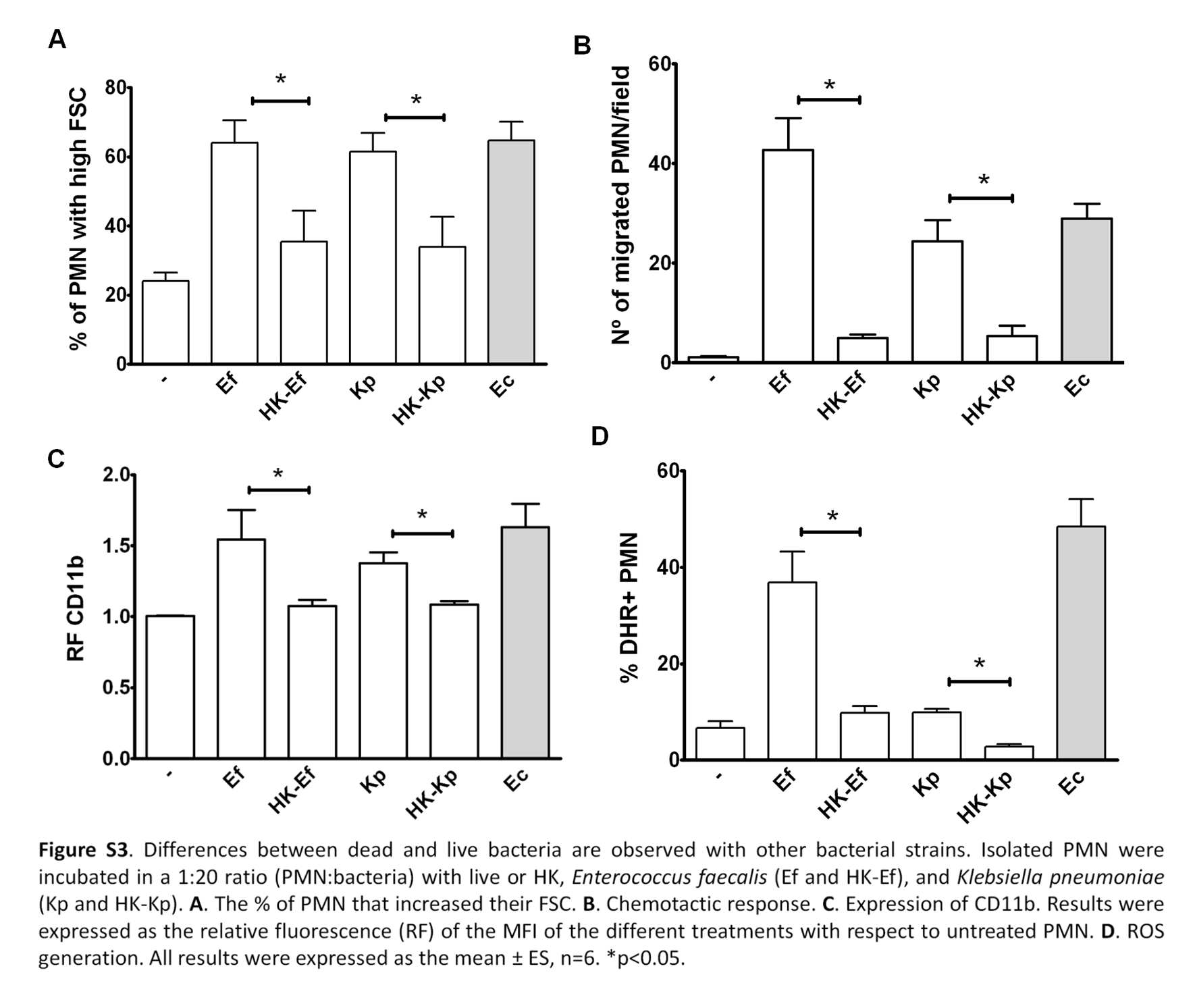

Supplement: Supplementary file 3 [file Image3.TIF]

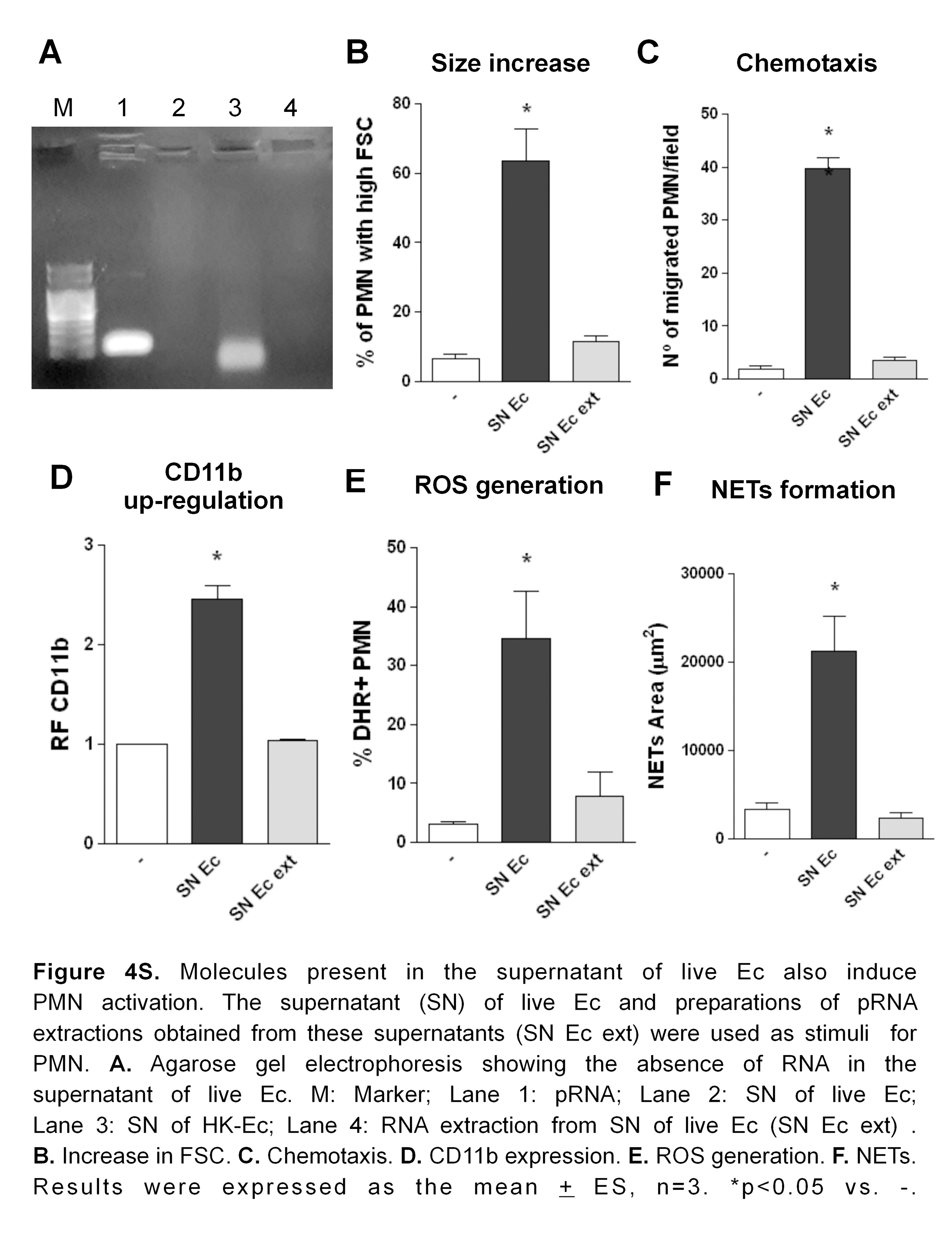

Supplement: Supplementary file 4 [file Image4.TIF]

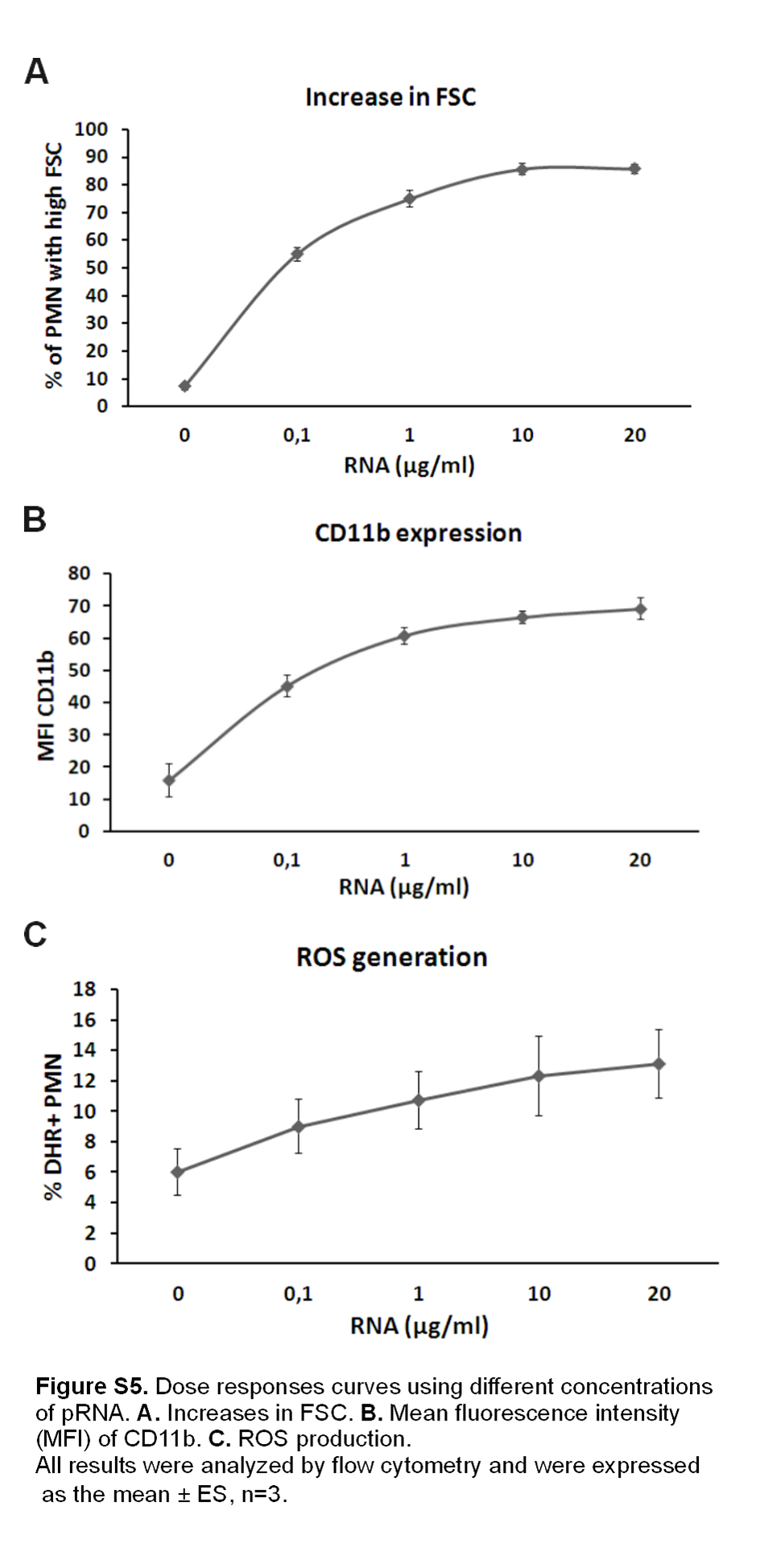

Supplement: Supplementary file 5 [file Image5.TIF]

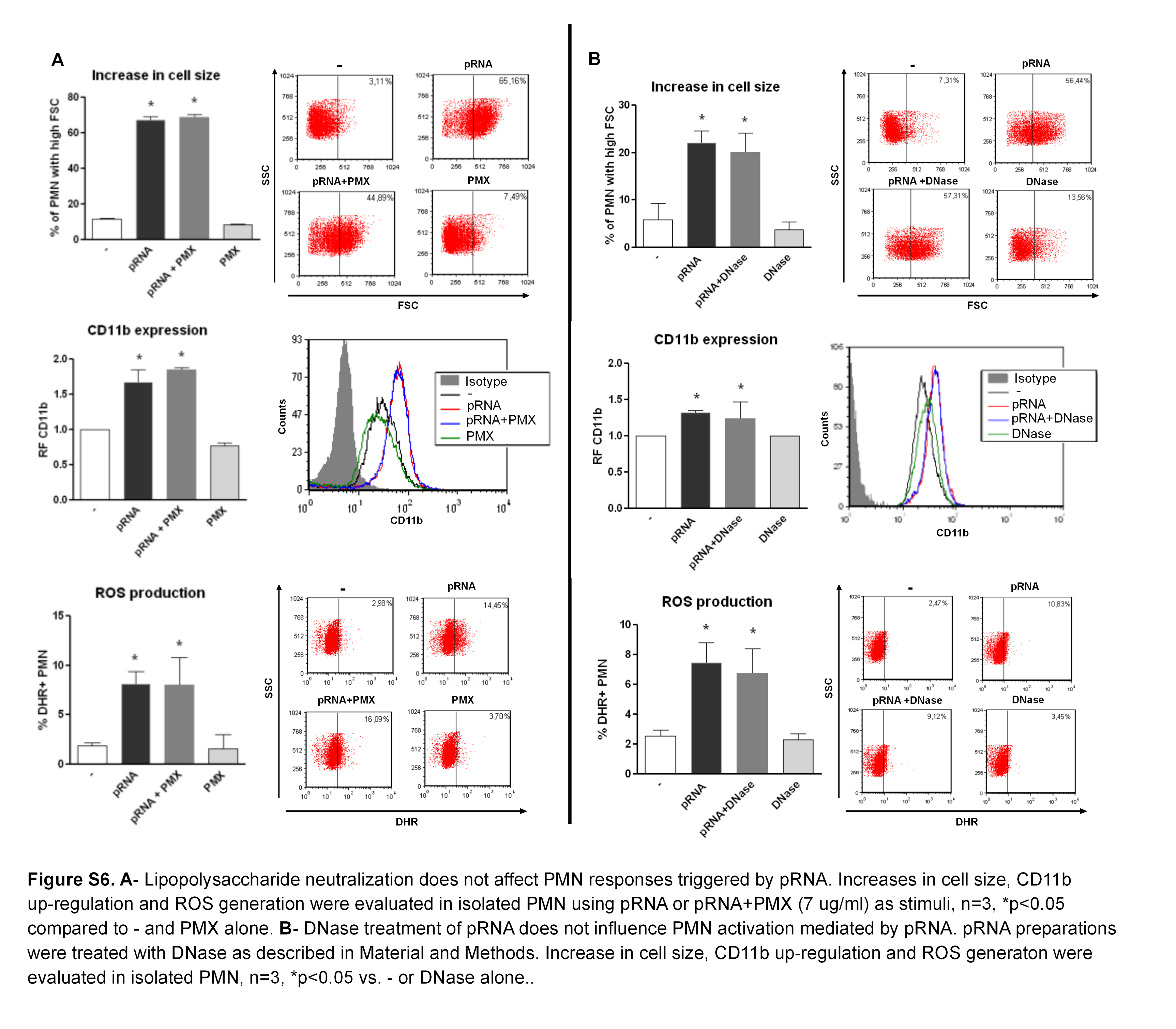

Supplement: Supplementary file 6 [file Image6.TIF]
